# Supplementary material for: Efficient Adsorption of Methylene Blue by Porous Biochar Derived from Soybean Dreg Using a One-Pot Synthesis Method
Source: Molecules. 2021 Jan 27;26(3):661. doi: 10.3390/molecules26030661 (PMC7865629; doi:10.3390/molecules26030661)
Supplement: Supplementary file 1 [file molecules-26-00661-s001.pdf]

Supplementary data

Efficient Adsorption of Methylene Blue by Porous Biochar Derived from Soybean Dreg Using a One-Pot Synthesis Method

Zhiwei Ying [1,2], Xinwei Chen [1,2], He Li [1,2,*], Xinqi Liu [1,2,*], Chi Zhang [1,2], Jian Zhang [1,2] and Guofu Yi [1,2]

1. National soybean processing industry technology innovation center, Beijing Technology and Business University (BTBU), Beijing 100048, China; yingzhiwei0906@163.com (Z.Y.), chenxinweilulu@163.com (X.C.); cauzhangchi@163.com (C.Z.), tsnpzhj@163.com (J.Z.), yiguofu0203@163.com (G.Y.)
2. Beijing Advanced Innovation Center for Food Nutrition and Human Health, Beijing Engineering and Technology Research Center of Food Additives, Beijing Technology and Business University (BTBU), Beijing 100048, China

* Correspondence: lihe@btbu.edu.cn (H.L.), liuxinqi@btbu.edu.cn (X.L.)

Figures:

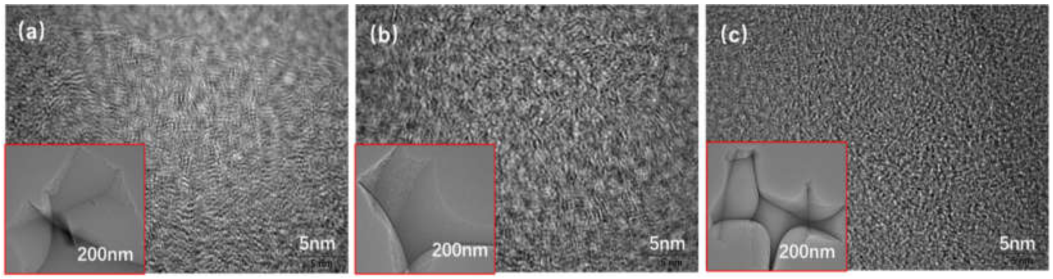

Figure S1. (a) TEM images of the SDB-K-2; (b) SDB-K-4; (c)SDB-K-5.

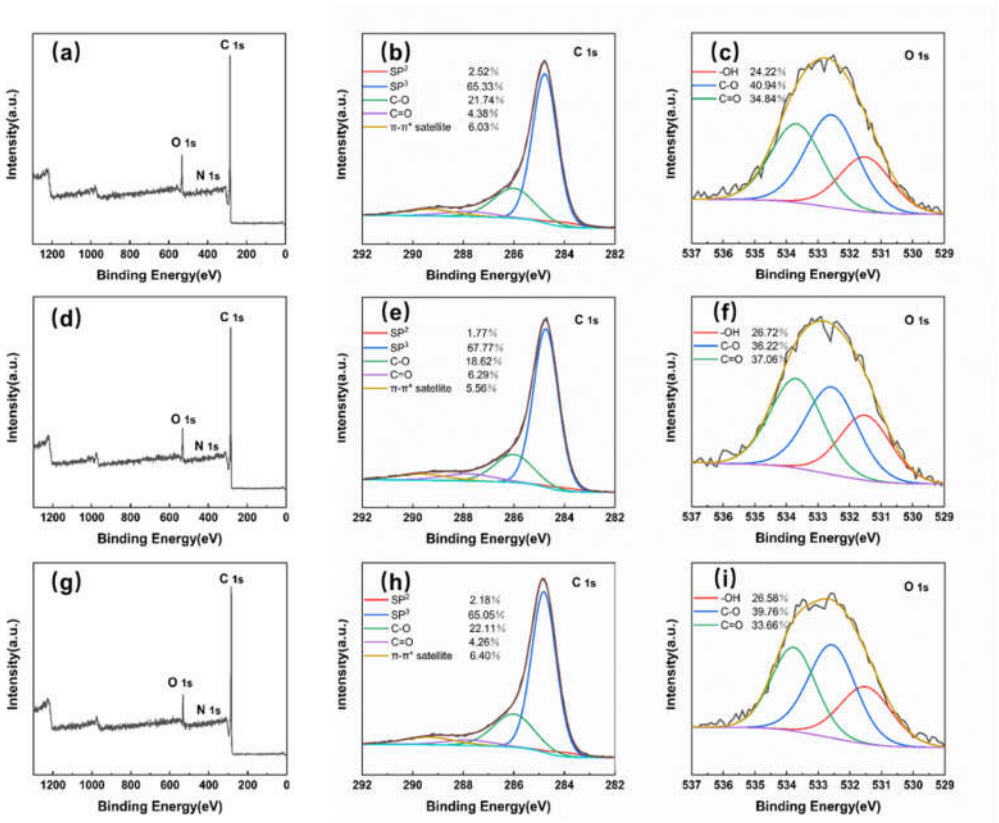

Figure S2. (a) XPS survey of SDB-K-2, (b) high-resolution spectra of C1s of SDB-K-2, and (c) high-resolution spectra of O1s for SDB-K-2. (d) XPS survey of SDB-K-4, (e) high-resolution spectra of C1s of SDB-K-4, and (f) high-resolution spectra of O1s for SDB-K-4. (g) XPS survey of SDB-K-5, (h) high-resolution spectra of C1s of SDB-K-5, and (i) high-resolution spectra of O1s for SDB-K-5.
